# Supplementary figures and images for: Large-Scale Preparation of Uniform Millet Bread-like Durable Benzoxazine-Phthalonitrile Foam with Outstanding Mechanical and Thermal Properties
Source: Polymers (Basel). 2022 Dec 10;14(24):5410. doi: 10.3390/polym14245410 (PMC9781014; doi:10.3390/polym14245410)

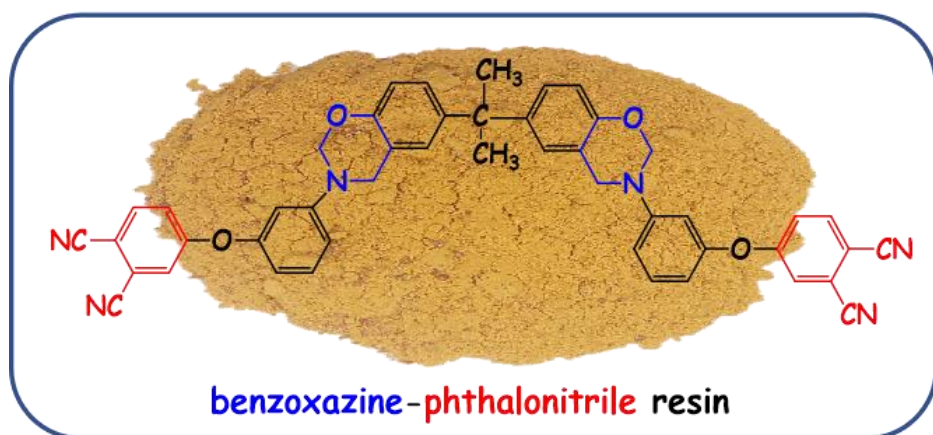

Figure S1 Chemical structure of the bi-functional benzoxazine-phthalonitrile resin

Supplement: Supplementary file 1 [file polymers-14-05410-s001.zip › polymers-2048080-supplementary.pdf]
